# Pacin, a novel bacteriocin from *Pseudomonas* strain 166, exhibits anti-MRSA activity with low toxicity and high therapeutic potential

**DOI:** 10.1128/spectrum.04198-25

**Published:** 2026-06-22

**Authors:** Yu Wang, Xiaojia Fu, Yunjia He, Yue Ma, Xin Meng, Yi Yang, Lingcong Kong, Haiyong Guo

**Affiliations:** 1 Jilin Normal University 47825, Siping, Jilin, China; 2 Jilin Agricultural University 85112, Changchun, Jilin, China; Pennsylvania State University, University Park, Pennsylvania, USA

**Keywords:** MRSA, bacteriocins, antibacterial activity, safety, antibacterial mechanism

## Abstract

The emergence and rapid spread of methicillin-resistant *Staphylococcus aureus* (MRSA) pose a significant threat to public health, underscoring the urgent need for the development of effective antibacterial agents. Bacteriocins, as highly promising antibacterial candidates, have garnered considerable attention in the scientific community. In this study, a novel bacteriocin, designated Pacin, was successfully purified from the fermentation broth of *Pseudomonas* strain 166. Pacin exhibited potent antibacterial activity against MRSA, with minimum inhibitory concentration (MIC) of 0.25 µg/mL. At concentrations up to 512 µg/mL, Pacin showed no significant cytotoxicity toward Vero or F81 cells and induced negligible hemolysis of rabbit red blood cells, with a hemolytic rate below 0.04%. In a murine model of MRSA-infected wounds, Pacin significantly promoted wound healing. In a peritonitis-sepsis model, mice treated with Pacin displayed no organ abnormalities and a marked reduction in bacterial burden. Further analyses, including cell membrane integrity assays, cell wall integrity assessments, scanning electron microscopy, and transmission electron microscopy, demonstrated that Pacin exerts its antibacterial effect by disrupting the bacterial cell membrane and cell wall structures, thereby inhibiting bacterial survival. Collectively, these findings establish Pacin as a highly effective antibiotic alternative for combating MRSA infections, with substantial potential for clinical application.

**IMPORTANCE:**

This study introduces Pacin, a novel bacteriocin isolated from *Pseudomonas* strain 166, as a potent therapeutic agent against MRSA. With the global rise of antibiotic resistance threatening public health, there is an urgent need for safe and effective alternatives to conventional drugs. Pacin demonstrates exceptional activity by effectively killing MRSA through membrane and cell wall disruption while showing no toxicity to mammalian cells or zebrafish embryos. Its stability under varying environmental conditions and shows efficacy in treating severe skin infections and sepsis in animal models highlight its clinical potential. These findings position Pacin as a promising next-generation antimicrobial agent capable of addressing critical gaps in current treatment strategies for life-threatening resistant infections.

## INTRODUCTION

Antimicrobial resistance (AMR) represents a formidable global public health challenge and is increasingly recognized as a critical “One Health” issue. The interconnected nature of human, animal, and environmental health domains facilitates the emergence, evolution, and global dissemination of antibiotic-resistant pathogens, thereby positioning AMR as a major threat to global health (1). Projections indicate that by 2050, multidrug-resistant bacteria could contribute to up to 10 million annual deaths due to resistance across a wide range of antibiotics (2, 3). A seminal 2022 study published in *The Lancet* estimated that in 2019, AMR was associated with approximately 4.95 million deaths across 204 countries and territories worldwide, with 1.27 million deaths directly attributable to resistant bacterial infections (4).

*Staphylococcus aureus* (*S. aureus*) is one of the most common bacterial pathogens worldwide, causing a substantial burden of skin and soft tissue infections each year, as well as numerous severe invasive infections. In the United States alone, *S. aureus* is responsible for approximately 20,000 deaths each year (5), a mortality toll that may surpass that of AIDS (6). The clinical management of *S. aureus* infections is particularly challenging due to the frequent emergence of antibiotic resistance, most notably MRSA, which significantly complicates treatment strategies.

First identified in 1961, MRSA has become a major public health concern worldwide. In many regions, including the United States, approximately 25% of *S. aureus* isolates are methicillin-resistant (7). MRSA is associated with a wide range of diseases, including septicemia, pneumonia and soft tissue infections, and is often accompanied by substantial mortality, rapid transmission, and significant healthcare costs (8, 9). Vancomycin has historically been the cornerstone of treatment for MRSA infections; however, recent declines in vancomycin susceptibility have exacerbated the public health challenge, underscoring the urgent need for alternative antimicrobial agents (10).

Bacteriocins, biologically active peptides or proteins produced by bacteria, are increasingly valued for their high specificity, favorable safety profiles, multifunctionality, environmental compatibility, and broad potential applications (11–14). Notably, bacteriocins can act synergistically with conventional antibiotics to enhance therapeutic efficacy against multidrug-resistant pathogens (15). Ongoing research has demonstrated that many bacteriocins exhibit potent inhibitory activity against both Gram-positive and Gram-negative bacteria, with excellent physicochemical stability, including high thermal tolerance and broad pH adaptability, which supports their utility across diverse environmental conditions. Advances in research and technology continue to uncover novel bacteriocins, providing a robust foundation for the development of next-generation antimicrobial agents.

In this study, we first evaluated the antibacterial activity of a novel bacteriocin, Pacin, against MRSA. We then investigated the effects of temperature, pH, and enzymatic treatments on its antibacterial activity under *in vitro*. Additionally, we assessed the *in vivo* and *in vitro* safety profiles and therapeutic efficacy of Pacin. Finally, we explored the antibacterial mechanism of action of Pacin to assess its potential as a safe and effective alternative to conventional antibiotics.

## MATERIALS AND METHODS

### Materials

The Alkaline Phosphatase Assay Kit, ATP Assay Kit, and Reactive Oxygen Species Assay Kit were obtained from Nanjing Jiancheng Bioengineering Institute (Nanjing, China). The SYTO9-PI Live/Dead Bacterial Staining Kit was purchased from Beijing Tianjingsha Gene Technology Co., Ltd. (Beijing, China). Sodium dehydroacetate, lipase, pepsin, trypsin, and alkaline protease were acquired from Pusitang Biotechnology Co., Ltd. The Cell Counting Kit-8, ammonium persulfate, LB broth, SDS-PAGE Preparation Kit, dialysis membranes, unstained protein marker, and BCA Protein Assay Kit were purchased from Sangon Biotech Co., Ltd. (Changchun, China). N-phenyl-1-naphthylamine (NPN) was obtained from Sigma-Aldrich (Shanghai, China). The Bacterial DNA Extraction Kit was supplied by Solarbio Life Sciences (Beijing, China). Mueller-Hinton Agar (MHA) and Mueller-Hinton Broth (MHB) were procured from Coolaber Technology Co., Ltd. (Beijing, China). The Calcein/PI Cell Viability/Cytotoxicity Assay Kit was obtained from Beyotime Biotechnology (Shanghai, China).

### Bacterial strains

*Pseudomonas* sp. strain 166 was obtained from the Laboratory of Pharmacology, Jilin Agricultural University (Changchun, China). MRSA was provided by the Microbiology Laboratory, Jilin Normal University (Siping, China).

### Antibacterial activity assay

The antibacterial activity was evaluated using the agar well diffusion method, as described previously (16). MRSA was cultured in MHB at 37°C until reaching an optical density (OD_600_) of 0.6. The culture was then diluted 1,000-fold, and 100 µL of the diluted bacterial suspension was spread onto Mueller-Hinton agar (MHA) plates. Wells were created in the agar using Oxford cups. *Pseudomonas* sp. strain 166 was cultivated at 16°C for 72 h, and the culture supernatant was obtained by centrifugation and concentrated 10-fold. Subsequently, 100 µL of the concentrated supernatant was added into the wells and incubated at 37°C for 24 h.

### Purification of bacteriocin Pacin

Bacteriocin Pacin was purified through a multi-step process involving ammonium sulfate precipitation, dextran gel chromatography (Sephadex G-75), and anion-exchange chromatography. Briefly, 1 mL of *Pseudomonas* sp. strain 166 in the logarithmic growth phase was inoculated into 300 mL of LB broth and incubated at 16°C for 72 h. The cell-free supernatant was obtained by centrifugation at 8,000 × *g* for 30 min at 4°C. Ammonium sulfate was incrementally added to the supernatant to precipitate proteins, followed by centrifugation at 8,000 × *g* for 10 min at 4°C to collect the precipitate. The precipitate was resuspended in phosphate-buffered saline (PBS) and dialyzed at 4°C for 12 h using a 3.5 kDa molecular weight cut-off (MWCO) membrane to remove small-molecular-weight impurities. Antimicrobial activity was assessed using the agar well diffusion method, as described previously (17).

Active fractions were subjected to Sephadex G-75 gel filtration chromatography, with elution performed using PBS at a flow rate of 0.5 mL/min. Fractions exhibiting antimicrobial activity against MRSA were collected and evaluated via the agar well diffusion method.

Further purification was conducted using Q-uSphere strong anion-exchange chromatography, following established protocols (18). Bound proteins were eluted stepwise with increasing NaCl concentrations. Active fractions were re-dialyzed at 4°C using a 3.5 kDa MWCO membrane to remove residual salts. Protein concentrations were quantified using the BCA Protein Assay Kit to prepare samples for subsequent experiments.

### Molecular mass determination

The molecular mass of purified bacteriocin Pacin was determined using sodium dodecyl sulfate–polyacrylamide gel electrophoresis (SDS-PAGE) under denaturing conditions (19). Purified Pacin was denatured by heating at 95°C for 5 min in a sample buffer containing 5% β-mercaptoethanol. Electrophoresis was performed on a 12% polyacrylamide gel, followed by staining with Coomassie Brilliant Blue R-250. The molecular weight of Pacin was estimated by comparing its electrophoretic mobility to that of a protein marker (10–250 kDa). Additionally, Pacin bands were cut out from Coomassie-stained SDS-PAGE gels and subjected to liquid chromatography–tandem mass spectrometry (LC-MS/MS) analysis at Sangon Biotech Co., Ltd. (Wuhan, China) for precise molecular weight confirmation.

### Antimicrobial activity assays

The antibacterial efficacy of Pacin was evaluated following the methodology described by Wang et al. (20). MRSA was cultured to an OD₆₀₀ of 0.5 and diluted with MHB to a concentration of 10⁶ CFU/mL. Aliquots (100 µL) of Pacin at various concentrations were combined with 100 µL of bacterial suspension in sterile 96-well microtiter plates and incubated at 37°C for 12 h. MICs were determined by measuring absorbance at 600 nm using a multimode microplate reader (Tecan, Switzerland). To determine the minimum bactericidal concentration (MBC), 100 µL from wells exhibiting no visible growth was spread onto MHA plates and incubated at 37°C for 24 h. The MBC was defined as the lowest concentration resulting in 99.9% reduction in viable bacteria, according to established criteria (21).

MRSA at a concentration of 10⁶ CFU/mL was exposed to various concentrations of Pacin, with untreated bacteria serving as the control. Bacterial growth was monitored every 4 h using a Bioscreen C Automated Growth Curve Analyzer (Bioscreen, Finland).

### Stability under enzymes, heat, and pH conditions

The stability of Pacin was evaluated against enzymatic, thermal, and pH challenges (16). For enzymatic stability, Pacin was incubated with trypsin, papain, lipase, alkaline protease, catalase, and pepsin (final concentration of 1 mg/mL ) at 37°C for 1 h. Thermal stability was assessed by exposing Pacin to temperatures of 40, 60, 80, or 100°C for 30 min. To evaluate pH stability, Pacin was incubated at pH values ranging from 2 to 10 for 1 h, followed by neutralization to pH 7. Untreated Pacin served as the control. Residual antimicrobial activity after each treatment was quantified using the agar well diffusion method.

### Hemolytic assay

The hemolytic activity of Pacin was assessed following the protocol by Wang et al. (20). Fresh erythrocytes from healthy rabbits were resuspended in sterile saline to a 1% (v/v) concentration. Erythrocyte suspensions were mixed with Pacin at concentrations ranging from 1 to 512 µg/mL and incubated at 37°C for 1 h. PBS and 0.1% Triton X-100 were used as negative and positive controls, respectively. After centrifugation at 1,000 × *g* for 10 min, supernatants were transferred to 96-well plates, and hemoglobin release was quantified at 570 nm using a multimode microplate reader (Tecan, Switzerland). Hemolysis was calculated using the formula:


$$Percent\;hemolysis=[(A-A_{buffer})\;/\;(A_{Triton\;X-100}-A_{buffer})]\times 100$$

where *A* is the absorbance of Pacin-treated samples, *A*_*buffer*_ is the absorbance of the saline control, and *A*_*Triton X*_*_-_*_*100*_ is the absorbance of a 0.1% Triton X-100-treated sample (100% hemolysis).

### Cytotoxicity assay

Cytotoxicity was evaluated using the Cell Counting Kit-8 (CCK-8) assay (22). Cells (15,000 per well) were seeded in 96-well plates and then treated with Pacin at concentrations ranging from 1 to 512 µg/mL for 6 h at 37°C in a 5% CO₂ atmosphere. Subsequently, 10 µL of CCK-8 solution was added to each well, followed by a 1-h incubation. Wells containing only medium and CCK-8 (no cells) served as blanks, while untreated cells served as controls. Absorbance was measured at 450 nm using a multimode microplate reader (Tecan, Switzerland). Cell viability was calculated as:


$$Cell\;viability(\%)=[A_{\left(treated\right)}-A_{\left(blank\right)}]\;/\;[A_{\left(control\right)}-A_{\left(blank\right)}]\times 100$$

where *A*_*(treated)*_ is the absorbance of Pacin-treated cells, *A*_*(blank)*_ is the absorbance of the blank well without cells, and *A*_*(control)*_ is the absorbance of untreated cells.

### Viability/cytotoxicity assay

Pacin’s cytotoxicity was further assessed using a Calcein/PI Cell Viability/Cytotoxicity Assay Kit, according to the protocol described by Deng et al. (23). Vero and F81 cells were seeded in 96-well plates at a density of 10⁴ cells per well and treated with Pacin (1 and 512 µg/mL) for 2 h. Untreated cells served as the negative control. Post-treatment, cells were washed three times with PBS and stained with calcein-AM and PI in the dark. Images were then captured using an inverted fluorescence microscope (Leica, Germany).

### Zebrafish embryo toxicity assay

Zebrafish embryo toxicity was evaluated using the method described by Liu et al.(24). At 48 h post-fertilization, zebrafish embryos were exposed to Pacin at concentrations ranging from 1/8 MIC to 4 MIC for 24 h at 28°C. A negative control (without Pacin) and a positive control (200 µg/mL sodium dehydroacetate) were included. Embryonic phenotypes were observed using an AXIO Zoom.V16 stereo microscope (ZEISS, Germany).

### *In vivo* toxicity assays

*In vivo* toxicity evaluations were conducted following established protocols (25). Kunming mice, aged 30 days and weighing approximately 20 g, were procured from the Jilin University Laboratory Animal Center, with six mice per experimental group. Mice received intraperitoneal injections of Pacin at doses of 0.25 mg/kg or 1 mg/kg, or an equivalent volume of saline, administered every 8 h for 7 consecutive days. Subsequently, mice were euthanized. Blood samples were obtained from the retro-orbital sinus and centrifuged to isolate serum for biochemical analysis. Organs were harvested and subjected to histopathological examination using hematoxylin and eosin (H&E) staining.

### Evaluation of Pacin on healing of MRSA-infected skin wounds in mice

The therapeutic efficacy of Pacin on MRSA-infected skin wounds was assessed as previously described (26). Briefly, 30-day-old Kunming mice (*n* = 24) were anesthetized, and an 8- mm-diameter full-thickness excisional wound was created on their dorsal surface. Each wound was inoculated with 100 µL of MRSA at a concentration of 10⁶ CFU/mL. Six hours post-inoculation, mice were randomly allocated to four groups (*n* = 6 per group) and treated topically with saline (control), Pacin at 0.25 mg/kg or 1 mg/kg, or vancomycin at 0.25 mg/kg, applied every 12 h for 7 days. Wound healing was monitored daily through photographic documentation. On day 7, wound surfaces were swabbed with sterile cotton, and bacterial counts were determined via plating on LB agar medium.

### *In vivo* peritonitis-sepsis model

To evaluate the therapeutic efficacy of Pacin in a peritonitis-sepsis model, we utilized 30-day-old Kunming mice, with an equal distribution of males and females. The model was established following previously reported methods (25). Mice were intraperitoneally injected with MRSA. One hour post-infection, mice were administered either 0.25 mg/kg or 1 mg/kg Pacin or PBS as a control. Twenty-four hours after infection, mice were euthanized, and tissue samples from the heart, liver, spleen, lung, and kidney were collected for histopathological analysis using H&E staining. For bacterial quantification, organ homogenates were serially diluted, and 100 µL of each dilution was plated onto agar medium, followed by overnight incubation at 37°C to determine bacterial burden.

### DNA retardation assays

DNA retardation assays were assessed using a gel retardation assay, as described previously (27). Briefly, 200 ng of DNA was incubated with Pacin at concentrations ranging from 0.16 to 256 µg/mL in 20 µL of binding buffer (composed of 10 mM Tris-HCl [pH 8.0], 1 mM dithiothreitol, 5% glycerol, 20 mM KCl, 1 mM EDTA, and 50 µg/mL bovine serum albumin). The reaction mixture was incubated at 37°C for 1 h. Subsequently, samples were analyzed by 1% agarose gel electrophoresis to evaluate DNA-Pacin complex formation.

### Cell membrane permeability assay

Membrane integrity was assessed following established protocols (28). Log-phase cultures of MRSA were harvested by centrifugation, washed thrice with PBS, and adjusted to an optical density (OD_600_) of 0.5. Cell suspensions were combined with Pacin at concentrations ranging from 1/8 MIC to 4 MIC in sterile 96-well plates and incubated at 37°C. Untreated cells (without Pacin) served as the negative control. After incubation, samples were centrifuged at 3,000 × *g* for 10 min, and the supernatant was transferred to a new 96-well plate. Propidium iodide (PI) was added to a final concentration of 10 µM, and samples were incubated at 37°C for 30 min. Fluorescence intensities were measured at excitation/emission wavelengths of 535 nm/615 nm using a microplate reader.

### Bacterial viability assay

Bacterial viability was analyzed as described by Zhu et al. (29). MRSA suspensions were adjusted to 10⁶ CFU/mL based on OD₆₀₀ measurements and incubated with Pacin at 1 × MIC for 2 h at 37°C. Untreated samples served as controls. Suspensions were centrifuged at 10,000 × *g* for 10 min, and the supernatant was discarded. The pellets were resuspended in 1 mL of 0.85% NaCl, mixed with 3 µL of a 1:1 SYTO9/PI dye solution, and incubated at room temperature for 15 min in the dark. Aliquots (10 µL) of stained suspensions were placed on microscope slides, covered with coverslips, and examined using an Olympus FV4000 laser scanning confocal microscope (Evident, Japan).

### Measurement of alkaline phosphatase (AKP) release

AKP leakage assay was performed according to Zhang et al. (30). MRSA cells were cultured to an OD₆₀₀ of 0.5, washed, resuspended in PBS, and adjusted to 10⁶ CFU/mL. A negative control group consisting of cells treated with PBS alone was included. For the assay, 30 µL of double-distilled water was added to blank wells, 30 µL of 0.1 mg/mL phenol standard solution to standard wells, and 30 µL of sample supernatant to assay wells. Subsequently, 50 µL of buffer and 50 µL of substrate solution were added to all wells.The 96-well plate was incubated at 37°C for 15 min, after which 150 µL of chromogenic reagent was added to each well. Absorbance was measured at 520 nm. Alkaline phosphatase (AKP) activity was calculated using the formula:


$$AKP\;activity=[(A_{assay}-A_{blank})/(A_{standard}-A_{blank})]\times C_{standard}\times 100\times N$$

where *C* standard is the phenol standard concentration (0.1 mg/mL) and *N* is the sample dilution factor.

### TEM and SEM characterization

Microscopic analysis of bacteria was performed according to Zhang et al. (30). For SEM analysis, MRSA cells were cultured to an OD₆₀₀ of 0.5 and adjusted to 10⁶ CFU/mL. The bacterial suspensions were treated with Pacin at MIC and 4 MIC for 2 h and subsequently fixed overnight at 4°C in 2.5% (w/v) glutaraldehyde. The fixed cells were dehydrated through a graded ethanol series (50%, 70%, 90%, and 100% for 10 min each, followed by 100% ethanol for 15 min, a 1:1 mixture of 100% ethanol and tert-butanol for 15 min, and anhydrous tert-butanol for 15 min). Samples were critical-point dried with liquid carbon dioxide, coated with gold-palladium, and observed using a Phenom XL G2 scanning electron microscope (Thermo Fisher Scientific, Netherlands).

For TEM, MRSA cells (10⁶ CFU/mL) were treated with Pacin for 2 h at 37°C, with PBS-treated cells as controls. Cells were harvested, washed three times with PBS, and fixed with 2.5% glutaraldehyde. Prepared samples were processed and analyzed at Jilin University.

### ATP determination

Adenosine triphosphate (ATP), a critical energy molecule, influences various cellular processes, with reduced levels often associated with apoptosis, necrosis, or toxic stress. Intracellular ATP levels in MRSA were quantified using an ATP Assay Kit, according to the protocol described by Zhang et al. (30). Following a 2-h treatment with Pacin (1/8 MIC to 4 MIC), cells were harvested by centrifugation, resuspended in 300 µL of double-distilled water, and lysed in a boiling water bath for 10 min. Cells treated with PBS alone served as a negative control to establish baseline ATP levels. The assay solution was added to a 96-well plate, incubated at room temperature for 5 min, and luminescence intensity was measured using a Tecan multimode microplate reader (Tecan, Switzerland).

### ROS measurement

Reactive oxygen species (ROS), including superoxide radicals, hydrogen peroxide, and their derivatives, regulate cellular processes such as growth, differentiation, and apoptosis. Intracellular ROS levels were measured according to the protocol described by Liu et al. (31). Log-phase MRSA cells were washed and resuspended in PBS to an OD_600_ of 0.5. The cell suspensions were incubated with 10 µM 2',7'-dichlorodihydrofluorescein diacetate (DCFH-DA) at 37°C for 30 min, washed thrice with PBS, and combined with Pacin (1/8 MIC to 4 MIC) in 96-well plates. Negative control wells contained no DCFH-DA, while positive control wells included both DCFH-DA and H₂O₂. Following an additional 30-min incubation at 37°C, fluorescence was measured at excitation/emission wavelengths of 488 nm/525 nm using a Tecan multifunctional microplate reader (Tecan, Switzerland).

### Statistical analysis

Data were analyzed using GraphPad Prism 9 and expressed as mean ± standard deviation. All experiments were performed independently with three biological replicates. Statistical significance was assessed using unpaired *t*-tests for comparisons between two groups, and one-way ANOVA followed by Tukey’s post hoc test for multiple group comparisons. A *P* value < 0.05 was considered statistically significant.

## RESULTS

### Antibacterial activity assay

The inhibitory effects of the fermentation supernatant from *Pseudomonas* sp. strain 166 against MRSA were presented in Fig. S1, with an inhibition zone diameters of 46 mm.

### Purification and molecular weight determination of Pacin

**Fig 1:**
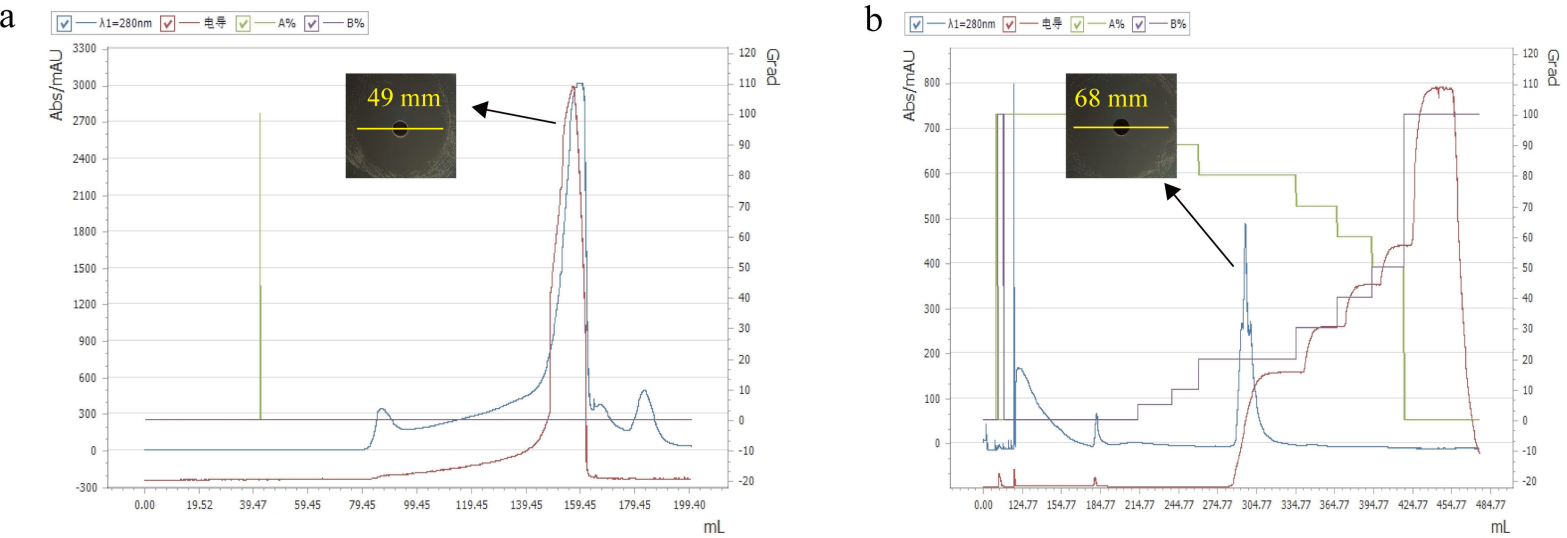
Purification of Pacin. (**a**) Dextran gel chromatography (Sephadex G-75). (**b**) Q-Sepharose column chromatography.

The bacteriocin, designated Pacin and produced by *Pseudomonas* sp. strain 166, was purified through a multi-step process involving ammonium sulfate precipitation, followed by Sephadex G-75 gel filtration chromatography and Q-uSphere strong anion-exchange chromatography. The elution profile from Sephadex G-75 gel filtration chromatography is presented in Fig. 1a. Further purification was conducted using anion-exchange chromatography, with active fractions eluted using a stepwise NaCl gradient. As shown in Fig. 1b, only the fraction corresponding to elution peak 3 exhibits antimicrobial activity as determined by the agar well diffusion assay.

**Fig 2:**
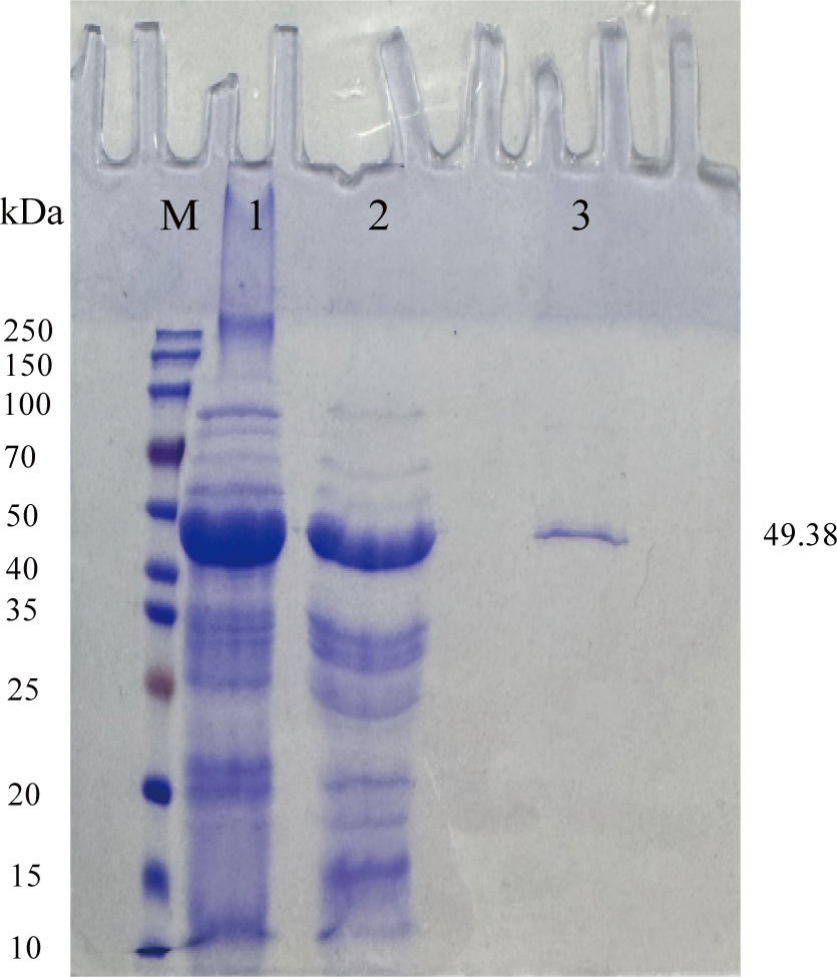
Molecular mass of Pacin was analyzed by SDS-PAGE. M, rainbow marker; 1, crude extracts; 2, Pacin-rich fractions from PrePack 16/600 Finedex 75 prep; 3, Pacin-rich fractions from Q-Sepharose column chromatography.

The molecular mass and homogeneity of purified Pacin were determined using sodium dodecyl sulfate–polyacrylamide gel electrophoresis (SDS-PAGE) and liquid chromatography–tandem mass spectrometry (LC-MS/MS). SDS-PAGE analysis revealed a single protein band with an estimated molecular weight of approximately 50 kDa (Fig. 2). LC-MS/MS analysis further confirmed the molecular mass of Pacin at 49.38 kDa (Fig. S2). These results indicate that purified Pacin is highly homogeneous, supporting its use in subsequent functional studies.

### Determination of the growth curve of MRSA after treatment with the Pacin

**Fig 3:**
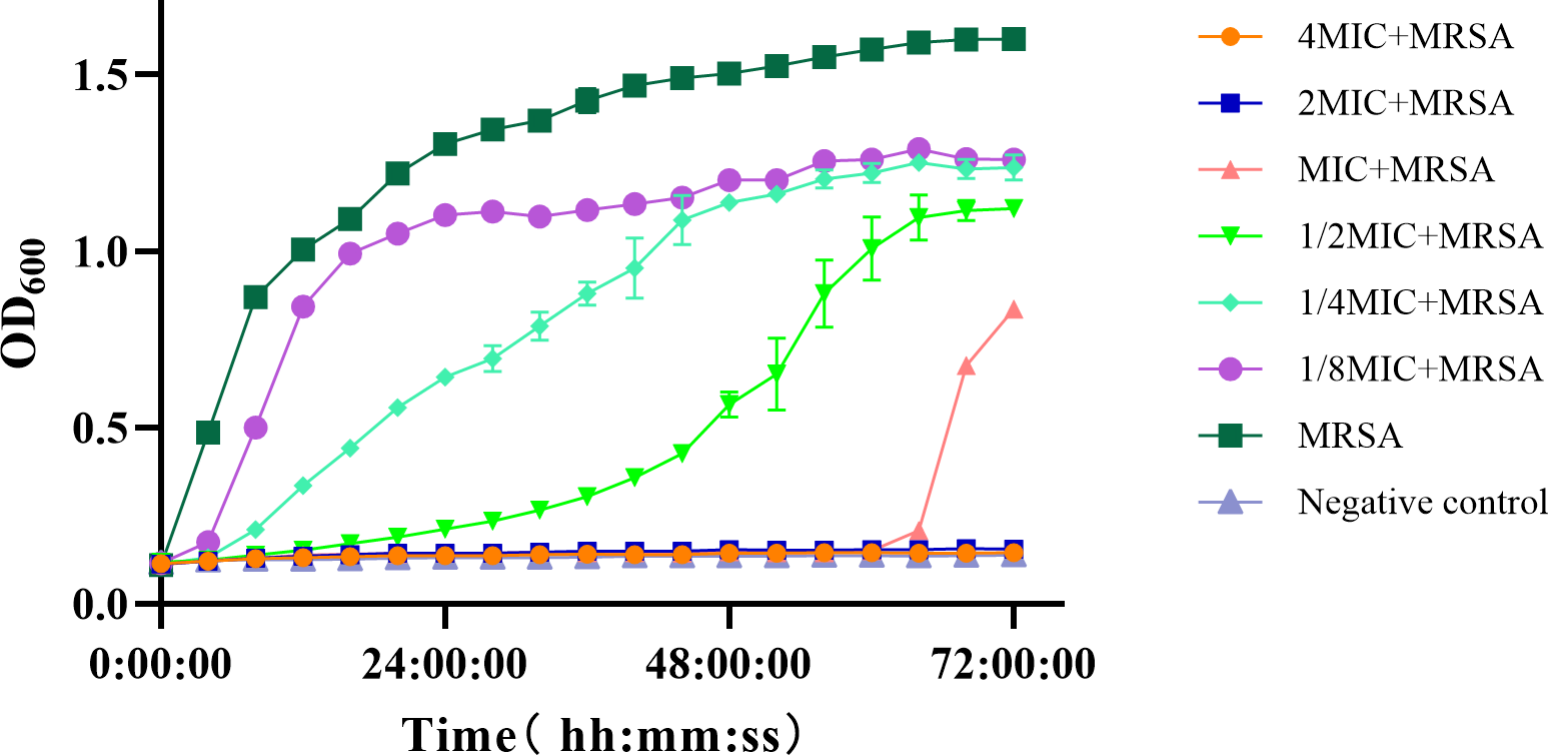
Growth curves of MRSA treated with Pacin.

The antibacterial activity of Pacin was evaluated *in vitro* by determining its MIC and constructing growth curves. Pacin exhibited significant antibacterial activity against MRSA, with an MIC value of 0.25 μg/mL. As shown in Fig. 3, at the MIC concentration, MRSA begin to grow at approximately 54 h. Moreover, under the action of 2 MIC and 4 MIC, MRSA showed no growth at 72 h.

### Stability assessment

**Fig 4:**
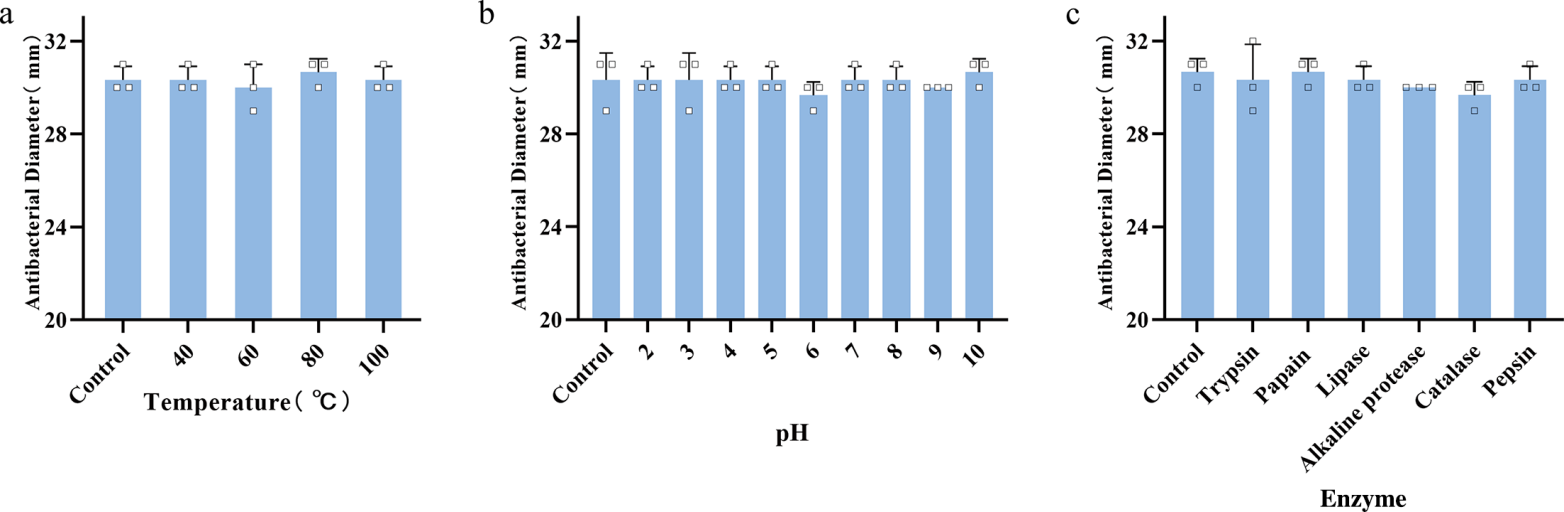
Stability analysis of Pacin. (**a**) Temperature stability of Pacin. (**b**) pH stability of Pacin. (**c**) Enzymatic stability of Pacin.

The stability of bacteriocins under varying environmental conditions is a critical factor for their potential applications (32). Pacin exhibits exceptional stability across a range of physicochemical conditions, as illustrated in Fig. 4. The bacteriocin retained significant antimicrobial activity following 1-h incubations at temperatures of 40°C, 60°C, 80°C, and 100°C. Similarly, Pacin maintained its activity across a pH range of 2–10 after 1-h exposures. Additionally, Pacin demonstrated remarkable resistance to enzymatic degradation, with no loss of antimicrobial activity observed after 1-h treatments with trypsin, papain, lipase, alkaline protease, catalase, and pepsin.

### Cytotoxicity and hemolytic activity

**Fig 5:**
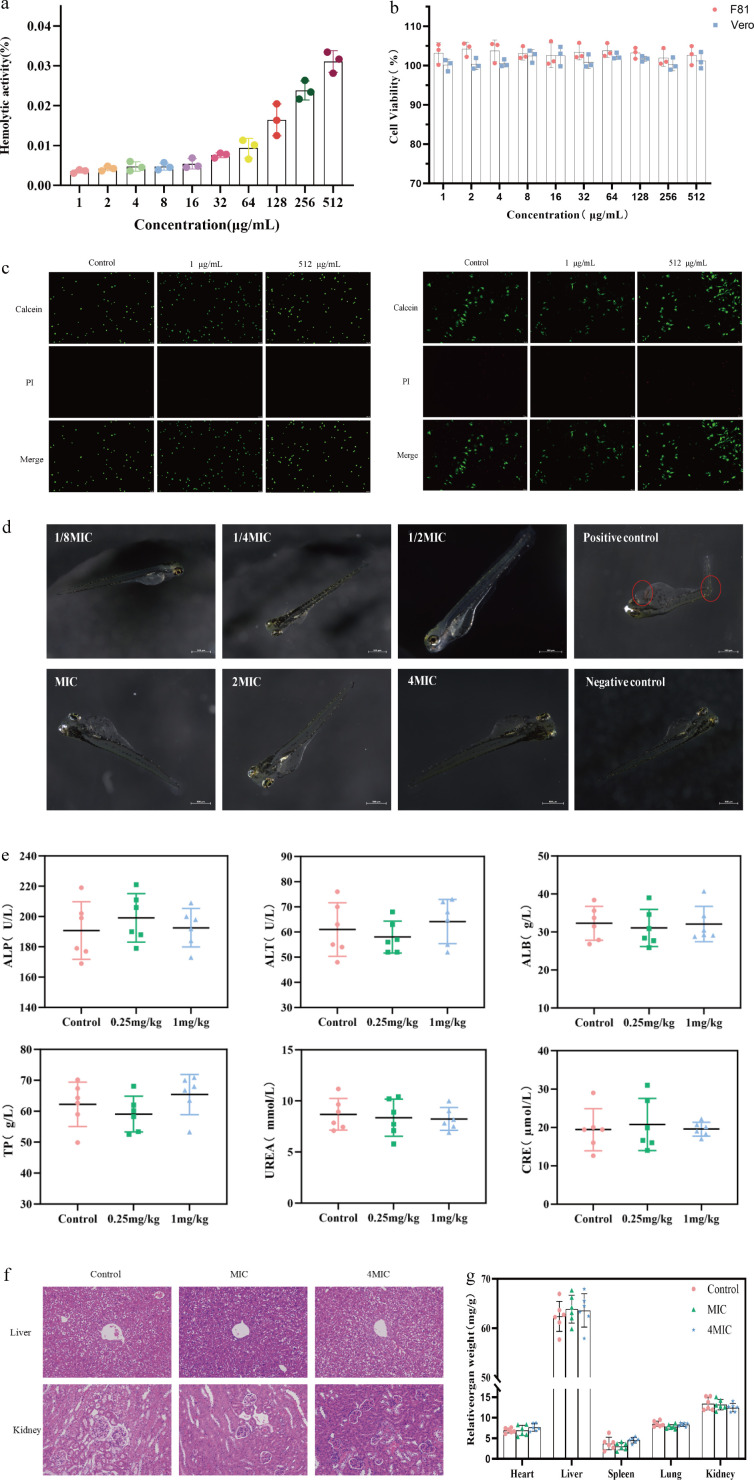
Safety analysis of Pacin. (**a**) Hemolytic activity of Pacin against rabbit red blood cells. (**b**) Cytotoxicity of Pacin against F81 cells (red) and Vero cells (blue). (**c**) Representative calcein-AM/PI staining images of F81 and Vero cells treated with Pacin. (**d**) Zebrafish embryo toxicity analysis of Pacin, embryo medium as a negative control, and sodium dehydroacetate (200 μg/mL) as a positive control. (**e**) Indices of hepatic and renal functions in Pacin-treated mice. (**f**) Histopathological analysis of liver and kidney tissues in mice after intraperitoneal injection of Pacin at different concentrations. (**g**) Organ index changes of heart, liver, spleen, lung, and kidney in mice injected with different concentrations of Pacin.

The biosafety evaluation of Pacin revealed exceptionally low hemolytic activity, with a hemolysis rate of less than 0.04% against rabbit red blood cells at a concentration of 512 μg/mL (Fig. 5a). Additionally, cytotoxicity assays conducted on Vero and F81 cell lines demonstrated that Pacin exhibited no cytotoxic effects across the tested concentration range (Fig. 5b). Further investigation via calcein-AM/propidium iodide (calcein-AM/PI) dual staining experiments indicated that, compared to the untreated control group, the number of viable cells remained largely unchanged following treatment with Pacin at concentrations of 1 μg/mL and 512 μg/mL (Fig. 5c).

### Toxicity assessment in zebrafish embryos

The developmental toxicity of Pacin was evaluated in zebrafish embryos exposed at 48 h post-fertilization to varying concentrations (1/8 MIC, 1/4 MIC, 1/2 MIC, MIC, 2 MIC, and 4 MIC). Pacin did not induce developmental toxicity in zebrafish embryos at the tested concentrations. In contrast, embryos in the positive control group exhibited significant abnormalities, including cardiac hyperemia and body curvature (Fig. 5d).

### Assessment of evaluating Pacin biosafety in mouse models

The biosafety profile of Pacin was investigated in mice through intraperitoneal administration. Hepatic function was evaluated by measuring serum alanine aminotransferase (ALT), alkaline phosphatase (ALP), albumin (ALB) and total protein (TP) levels. Nephrotoxicity was assessed by measuring serum urea (UREA) and creatinine (CRE) concentrations. No statistically significant elevations in these biomarkers were observed, indicating that Pacin does not adversely affect hepatic or renal function (Fig. 5e). Histopathological examination of liver and kidney tissues revealed no discernible pathological alterations in Pacin-treated mice compared to the control group (Fig. 5f). Furthermore, organ weight indices (heart, liver, spleen, lung, and kidney) exhibited no significant differences between Pacin-treated and control mice (Fig. 5g).

### *In vivo* antibacterial and wound-healing effects

**Fig 6:**
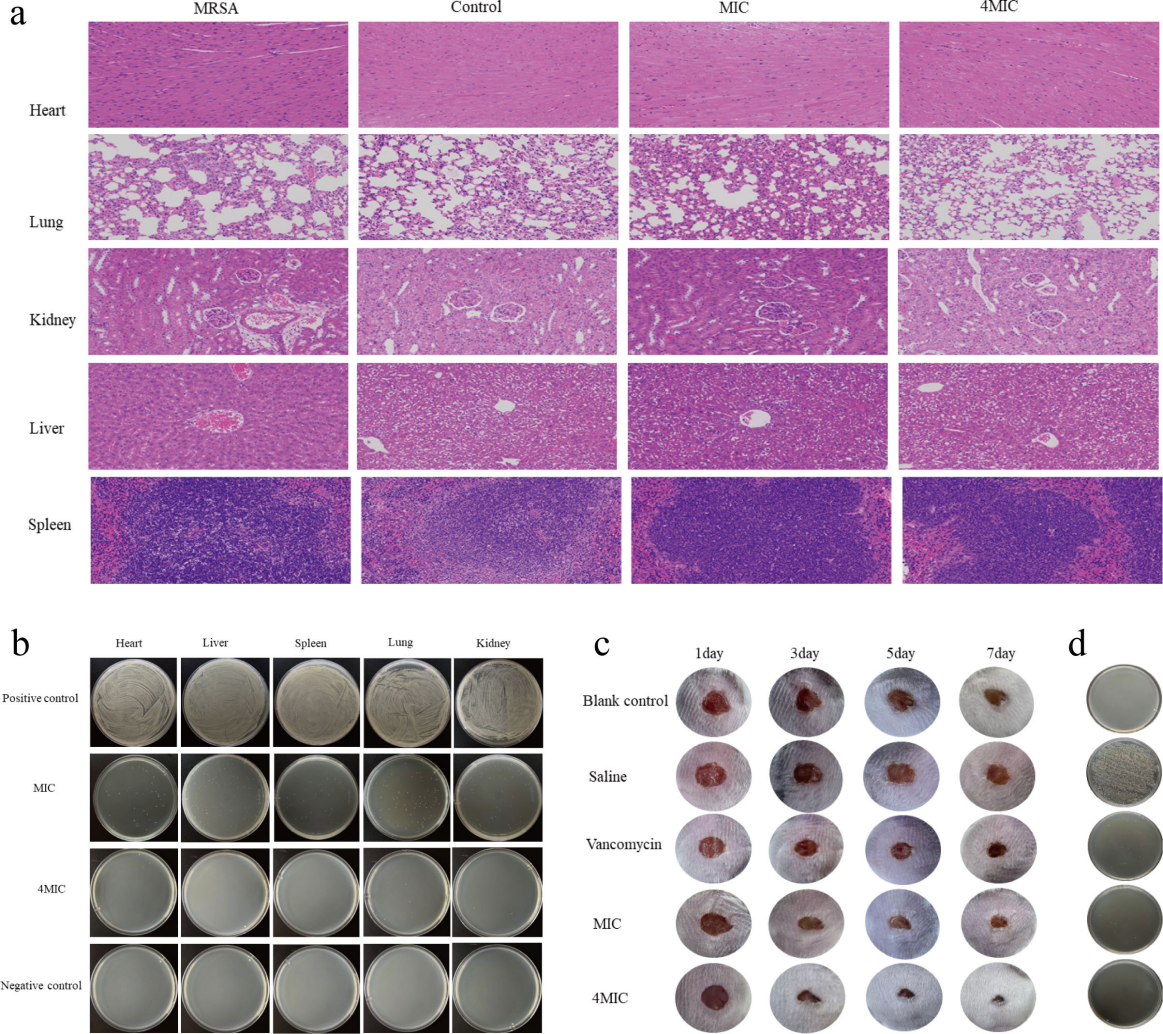
Pacin efficacy measurements. (**a**) Histopathological morphology of tissues following the administration of Pacin to mice with sepsis. (**b**) Analysis of organ bacterial load in sepsis model mice treated with Pacin. (**c**) Photographs of mouse wounds (control group, and groups treated with saline, MIC Pacin, 4 MIC Pacin, and vancomycin, respectively) during the treatment process on days 1, 3, 5, and 7. (**d**) Wound surface bacteria were cultured on agar plates on days 1, 3, 5, and 7 of treatment.

The therapeutic potential of Pacin was assessed in a murine model of MRSA-induced peritonitis-sepsis. Mice received intraperitoneal injections of Pacin at doses of 0.25 mg/kg or 1 mg/kg 1 h post-infection, with saline-treated mice serving as controls. H&E staining revealed pronounced hemorrhage and extensive inflammatory cell infiltration in the organs of saline-treated mice, whereas these pathological features were substantially attenuated or absent in the Pacin-treated group (Fig. 6a). Moreover, bacterial loads in the heart, liver, spleen, lung, and kidney were significantly reduced in the Pacin-treated group compared to controls (Fig. 6b). A positive correlation was observed between bacterial load and the degree of tissue damage, highlighting Pacin’s efficacy in reducing bacterial burden and mitigating associated histopathological changes in this peritonitis-sepsis model.

The therapeutic efficacy of Pacin in promoting wound healing was further evaluated in a murine model of bacterial infection. By day 7 post-treatment, significant differences in wound closure were observed across experimental groups (Fig. 6c). Compared to the saline and vancomycin control groups, the Pacin-treated group demonstrated significantly enhanced wound healing. Notably, wounds treated with Pacin at a concentration of 4 MIC exhibited near-complete closure. Additionally, a marked reduction in MRSA burden at the wound site was observed following Pacin treatment (Fig. 6d). These findings indicate that Pacin accelerates wound healing and reduces bacterial burden in infected wounds.

### DNA-binding assay

**Fig 7:**
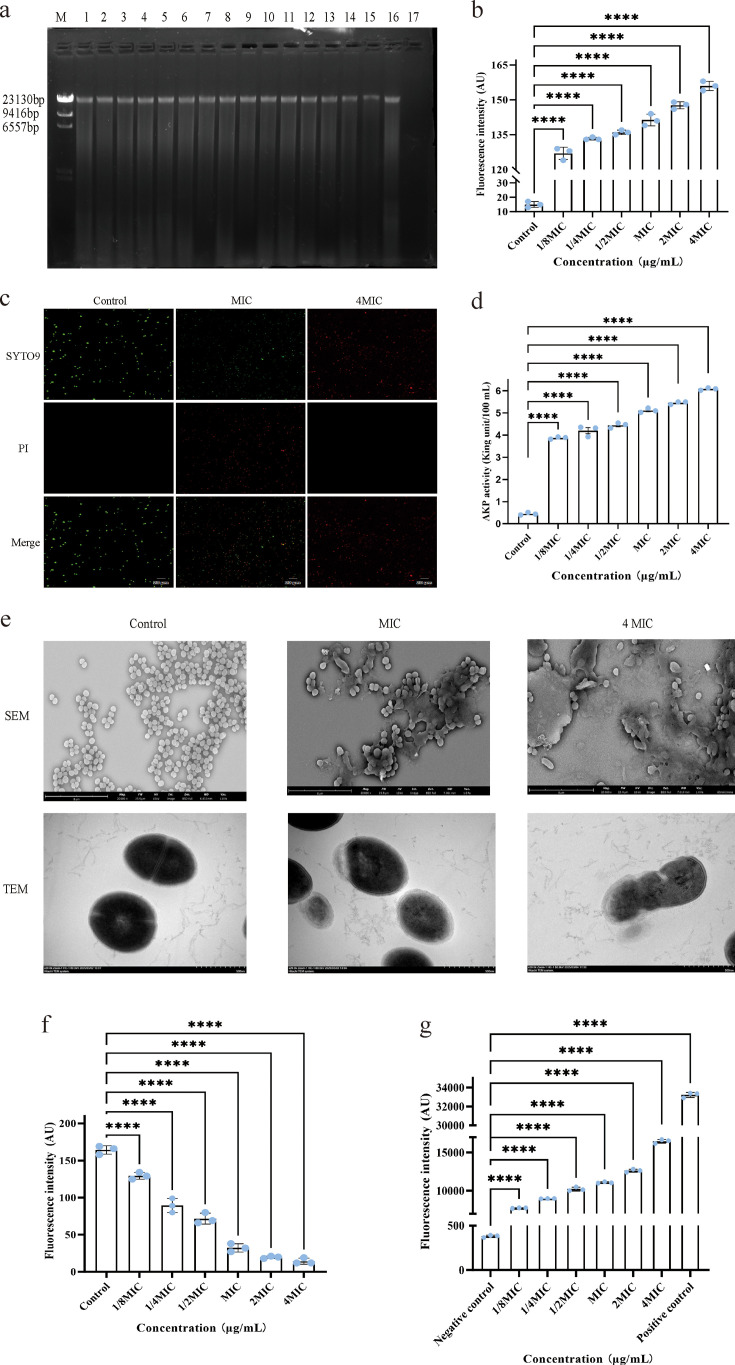
Inhibitory mechanism of Pacin against MRSA. (**a**) Effect of different concentrations of Pacin on DNA migration of MRSA. M: λ/HindIII DNA Marker; 1–15: DNA with 0.015625 μg/mL ~256 µg/mL of Pacin; 16: genomic DNA alone; 17: Pacin alone. (**b**) Changes in membrane permeability of MRSA cells after Pacin treatment. (**c**) Survival of MRSA after treatment with different concentrations of Pacin. (**d**) Effect of Pacin treatment on MRSA AKP release. (**e**) Morphological changes of MRSA treated with Pacin visualized with SEM and TEM. (**f**) Changes in intracellular ATP levels in MRSA after Pacin treatment. (**g**) Accumulation of ROS in Pacin-treated MRSA. Data are presented as mean ± SD. ****P < 0.0001.

The interaction between Pacin and bacterial DNA was investigated using an electrophoretic mobility shift assay. Pacin concentrations ranging from 0.015625 μg/mL to 256 μg/mL were tested. As shown in Fig. 7a, no inhibition of DNA migration is observed at any tested concentration, indicating that Pacin does not significantly interact with bacterial DNA under these conditions.

### Cell membrane permeability assay

PI is a fluorescent dye commonly employed to evaluate cell or bacterial viability, penetrating only the membranes of compromised cells and emitting red fluorescence. In this study, cell membrane permeability was assessed by measuring PI fluorescence intensity. Compared to the control group, fluorescence intensity increased with rising Pacin concentrations (Fig. 7b), indicating that Pacin enhances bacterial cell membrane permeability.

### Live/dead bacterial staining analysis

To elucidate the bactericidal mechanism of Pacin, live/dead staining was performed using SYTO9 and PI fluorescent dyes. SYTO9 penetrates all bacterial cell membranes, preferentially staining viable cells green under fluorescence microscopy, while PI selectively enters cells with compromised membranes, binds to nucleic acids, and stains dead cells red. This dual-staining approach enables clear differentiation between viable and non-viable bacteria, facilitating accurate assessment of Pacin’s bactericidal activity. Results indicated that, at a concentration of MIC, approximately 50% of MRSA cells were non-viable within 2 h. At 4 MIC, complete bacterial eradication was observed (Fig. 7c). These data suggest that Pacin primarily exerts its antibacterial effect by disrupting cell membrane structure and function.

### Evaluation of AKP release

Alkaline phosphatase (AKP), an enzyme located between the cell wall and membrane, serves as a marker for cell wall integrity, as its extracellular release indicates cell wall damage (30). The effect of Pacin on MRSA was assessed by measuring extracellular AKP levels. As shown in Fig. 7d, a significant increase in AKP release is observed in Pacin-treated cells compared with untreated controls across various concentrations. These results suggest that Pacin exerts its antibacterial activity, at least in part, by disrupting the bacterial cell wall.

### Ultrastructural analysis of MRSA

The effects of Pacin on the morphology and ultrastructure of MRSA were examined using SEM and TEM (Fig. 7e). SEM observations revealed that untreated bacteria possessed intact, regular, and sturdy cell structures. At Pacin’s MIC, cells showed notable deformation, with some displaying fragmentation and leakage of internal contents. At 4 MIC, a significant change in cell shape was observed, with a considerable increase in fragmented cells and widespread cell breakdown.

TEM results supported these findings. Compared to untreated controls, treatment with MIC Pacin induced distinct changes in cell membrane structure and internal organization. At 4 MIC, extensive damage to cell wall and membrane integrity was observed, along with significant leakage of cellular contents. These results highlight Pacin’s ability to disrupt bacterial cell wall and membrane integrity.

### Intracellular ATP levels and ROS production

Pacin’s effects on cellular ATP levels and ROS production in MRSA were characterized. ATP levels exhibited a dose-dependent decrease following Pacin treatment (Fig. 7f). Simultaneously, Pacin induced significant ROS accumulation, causing cells to undergo oxidative stress (Fig. 7g). This finding aligns with previous reports showing that ROS-mediated peroxidation of membrane phospholipids disrupts membrane integrity and fluidity, thereby promoting intracellular leakage.

## DISCUSSION

The escalating prevalence of antibiotic resistance poses a formidable challenge to global healthcare systems, as numerous bacterial pathogens develop resistance mechanisms that undermine the efficacy of conventional antibiotic therapies, complicating the management of infectious diseases. Consequently, the pursuit of novel antimicrobial agents exhibiting potent antimicrobial activity, high stability, and low toxicity has emerged as a critical research priority. Bacteriocins, in particular, have garnered significant interest due to their robust antimicrobial efficacy (33), broad-spectrum activity (34), and high stability and safety during prolonged use (35). In this study, we purified the bacteriocin Pacin through a three-step purification protocol and systematically evaluated its antimicrobial activity, stability, safety, and mechanism of action. Furthermore, we assessed its therapeutic potential and efficacy in MRSA infections to determine its suitability for clinical applications.

The purification of bacteriocins from fermentation broth represents a critical yet intricate process (36). In this study, we employed ammonium sulfate precipitation as the initial step to purify the fermentation broth derived from *Pseudomonas* sp. strain 166. Given the limitations of this method in achieving sufficient purity, we implemented a multi-stage chromatographic approach. Specifically, dextran gel chromatography was coupled with Q-uSphere strong anion-exchange chromatography to enhance separation and purification efficiency. This strategy aligns with established methodologies, such as those reported by Helber et al., who utilized ion exchange and gel filtration chromatography for the effective isolation of bromelain from natural sources (37). The homogeneity of Pacin was subsequently validated through SDS-PAGE and LC-MS/MS analyses. These findings not only confirmed the efficacy of the purification process but also provided a robust foundation for subsequent functional characterization and therapeutic application studies.

Bacteriocins have attracted substantial interest in fundamental and applied research due to their antimicrobial efficacy. Existing literature indicates that the MICs of most bacteriocins against bacterial strains typically range from 5 to 100 µg/mL (38). In this study, Pacin exhibited robust antimicrobial activity against MRSA, with an MIC value of 0.25 µg/mL. These values demonstrate superior potency compared to previously characterized bacteriocins, including PA996 (39), PA166 (40), and Pkmh (41), and notably surpass the efficacy of bacteriocins active against MRSA, including BMP32r (42), BM1300 (43), and CB6-C (30). Given its exceptional antimicrobial performance, Pacin holds considerable promise for applications in food preservation and medical therapeutics, offering a potent tool to address challenges posed by bacterial infections.

Beyond antimicrobial activity, bacteriocins are recognized for their resilience to enzymatic degradation and stability across a range of temperatures and pH conditions (44). These attributes—thermal stability, resistance to enzymatic breakdown, and tolerance to pH variations—are critical for their practical utility (32). Pacin demonstrated commendable stability, maintaining its activity under diverse temperature and pH conditions. This stability aligns with characteristics observed in bacteriocins produced by *Lactobacillus acidophilus* and *Lactobacillus bulgaricus* (45), as well as Bac10307 (46) and LPL-1 (47). For instance, bacteriocins from *Lactobacillus murinus* AU06 retained over 60% activity after 30 min at 60°C, while L23 from *Lactobacillus plantarum* LPL-1 preserved 90% activity at pH values below 7.0 (47). Furthermore, Pacin exhibited resistance to proteolytic enzymes, with no loss of antimicrobial activity following 1-h exposure to trypsin, pepsin, or papain, akin to the enzyme stability observed in NKR-5-3B (48). This robust stability under complex environmental conditions underpins Pacin’s potential for practical applications.

The safety of bacteriocins is of paramount importance as it is directly related to their use in areas such as food preservation and medical therapy. Ensuring the safety of bacteriocins mitigates adverse effects on human health, reduces potential harm to the environment, and ensures their efficacy and reliability in practical applications as natural preservatives or alternatives to antibiotics. Several studies have shown that certain bacteriocins have some cytotoxicity at high concentrations (49–51). In the present study, Pacin at a concentration of 512 μg/mL showed no toxicity to Vero or F81 cells, and its hemolysis rate of erythrocytes was less than 0.04%. To further evaluate its safety, Pacin was subjected to a zebrafish embryo acute toxicity assay, a well-established model for toxicological studies due to its sensitivity and ability to bridge *in vitro* and mammalian models (52, 53). This model facilitates the detection of contaminants, environmental monitoring, and assessment of toxicity, teratogenicity, and mechanisms of action (54). Exposure of zebrafish embryos to varying concentrations of Pacin resulted in no observable developmental abnormalities, indicating that Pacin did not exert overt toxic effects on embryonic development under the tested conditions. Additionally, hepatorenal safety, a key factor for clinical translation, was assessed via intraperitoneal administration of Pacin in mice. No significant elevations were observed in serum biomarkers (ALT, ALP, ALB, TP, UREA, CRE), and histopathological analyses revealed no pathological changes in liver or kidney tissues, suggesting negligible hepatic or renal toxicity. Comparable findings were reported for Plantaricin E and F, which exhibited normal liver and kidney morphology (55), and for bacteriocins TSU4 and pediocin N6, which showed no physiological abnormalities following oral administration (56, 57). Furthermore, major organ indices (heart, liver, spleen, lung, kidney) showed no significant differences between Pacin-treated and control groups. These results collectively underscore Pacin’s ability to maintain physiological homeostasis and its favorable biosafety profile, positioning it as a promising candidate for further development in biomedical and food safety applications.

The therapeutic potential of bacteriocins is key to their clinical translation as antimicrobial agents. Given that *S. aureus* is implicated in a range of severe pathologies, including cutaneous infections, peritonitis, and sepsis (58, 59), we developed murine models of cutaneous wound infection and peritonitis-sepsis to assess the therapeutic efficacy of Pacin. In the cutaneous wound infection model, Pacin treatment significantly reduced bacterial loads within the wounds, leading to markedly accelerated healing compared to the vancomycin-treated group. This enhanced healing may be attributed to diminished bacterial burdens, which likely alleviate impediments to tissue repair (42). In the peritonitis-sepsis model, Pacin significantly decreased bacterial counts in major organs of infected mice. Considering the established correlation between tissue damage and bacterial load (60), histopathological analysis using H&E staining revealed that Pacin-treated mice exhibited reduced tissue damage, with organ structures remaining relatively intact and a notable reduction in pathological features such as hemorrhage and inflammatory cell infiltration. These findings indicate that Pacin exerts superior therapeutic effects in promoting wound healing and mitigating peritonitis and sepsis.

Elucidating the mechanisms of action of bacteriocins, which represent a promising alternative to conventional antibiotics, is of paramount importance. Bacteriocins primarily exert their antimicrobial effects through mechanisms such as inhibition of protein synthesis, disruption of cell wall biosynthesis, perturbation of the cell envelope, and suppression of gene expression (61–63). In this study, we investigated Pacin’s mode of action by evaluating its effects on DNA binding, cell membrane permeability, and cell wall integrity. Our results demonstrate that Pacin primarily exerts its bactericidal activity by disrupting both cell wall and cell membrane integrity. This mechanism aligns with findings reported for other bacteriocins, including Angicin (64), plantaricin FB-2 (65), and LSB1 (66), all of which target cell membrane integrity. Yajuan et al. have noted that altering bacterial cell membrane permeability is a principal mechanism by which bacteriocins elicit their antimicrobial effects, as membrane disruption precludes bacteria from maintaining essential physiological functions (65). To further characterize cellular damage, SEM and TEM analyses revealed that Pacin-treated MRSA exhibited severe cellular damage, including cell wall disruption, membrane rupture, efflux of intracellular contents, and cell lysis. To corroborate these observations, a live/dead bacterial staining assay was performed using SYTO9, which stains all bacterial cells and exhibits enhanced fluorescence in viable cells, and PI, which selectively penetrates compromised membranes to bind nucleic acids. The results indicated that Pacin treatment resulted in a predominance of red-stained, non-viable cells, with the proportion of dead cells increasing in a dose-dependent manner. Additionally, Pacin-induced membrane disruption was associated with membrane depolarization, ATP leakage, and accumulation of ROS.

This finding indicates that Pacin treatment compromises the integrity of the MRSA cell membrane and cell wall. Wei et al. propose that membrane depolarization may result from potassium ion efflux caused by membrane damage, which subsequently disrupts proton dynamics (67). ATP serves as a critical marker of cell viability, with its extracellular release traditionally attributed to cellular disruption (68). Elevated ROS levels may act as a mediator of cell death, potentially arising from impaired electron transport in the respiratory chain due to damaged cell membranes (69). Our results demonstrate that Pacin disrupts the structural integrity of the cell membrane and cell wall, resulting in the consequent leakage of intracellular ATP into the extracellular space. Additionally, ROS accumulation may contribute to oxidative stress and potential effects on intracellular macromolecules. These cumulative effects ultimately may impair essential cellular processes, including metabolism, biosynthesis, and energy transduction, resulting in cell death. This discovery provides critical insights into the mechanism of action of Pacin and lays a foundation for developing novel therapeutic strategies targeting MRSA-associated diseases.

### Conclusion

In this study, we isolated and characterized Pacin, a novel bacteriocin produced by *Pseudomonas* sp. strain 166. Pacin exhibited potent inhibitory activity against MRSA *in vitro*, and mechanistic studies suggest that disruption of cell membrane integrity contributes to its antimicrobial action. Notably, Pacin retained full activity across a range of pH values and temperatures, demonstrating robust functional stability under physiologically and industrially relevant conditions. Importantly, no detectable cytotoxicity was observed in mammalian cells at concentrations that effectively inhibited MRSA. These combined attributes, including strong anti-MRSA activity, environmental resilience, and a favorable safety profile, support the potential of Pacin for applications in clinical antimicrobial therapy and food biopreservation. Future studies will focus on structural elucidation and *in vivo* efficacy evaluation to advance its translational development.

## SUPPLEMENTAL MATERIAL

Fig. S1 spectrum.04198-25-s0001.tif Antibacterial activity of crude extracts against MRSA.

Fig. S2 spectrum.04198-25-s0002.tif Partial MS/MS spectrum of Pacin.

Supplemental material spectrum.04198-25-s0003.docx Supplemental figure legends.

ASM does not own the copyrights to Supplemental Material that may be linked to, or accessed through, an article. The authors have granted ASM a non-exclusive, world-wide license to publish the Supplemental Material files. Please contact the corresponding author directly for reuse.
