# Supplementary material for: Pacin, a novel bacteriocin from Pseudomonas strain 166, exhibits anti-MRSA activity with low toxicity and high therapeutic potential
Source: Microbiol Spectr. 2026 Jun 22;14(8):e04198-25. doi: 10.1128/spectrum.04198-25 (PMC13435688; doi:10.1128/spectrum.04198-25)
Supplement: Supplementary file 1 [file spectrum.04198-25-s0003.docx]

Figure S1. Antibacterial activity of crude extracts against MRSA.

Figure S2. Partial MS/MS spectrum of Pacin.
